# Functional somatic syndromes are associated with inferior outcomes and increased complications after hip and knee arthroplasty: a systematic review

**DOI:** 10.1186/s42836-023-00223-1

**Published:** 2024-01-03

**Authors:** Raisa Masood, Krishna Mandalia, Nicholas R. Pagani, Michael A. Moverman, Richard N. Puzzitiello, Mariano E. Menendez, Matthew J. Salzler

**Affiliations:** 1 Department of Orthopaedic Surgery, Tufts Medical Center, Biewand Building, 7th Floor, 800 Washington St., Box 306, Boston, MA 02111 USA; 2 Tufts University School of Medicine, Boston, MA 02111 USA; 3 Oregon Shoulder Institute, Medford, OR 97504 USA

**Keywords:** Functional somatic syndromes, TKA, THA, UKA, PROMs

## Abstract

**Background:**

Functional somatic syndromes (FSSs), defined as chronic physical symptoms with no identifiable organic cause, may impact results after hip and knee arthroplasty. The purpose of this study was to perform a systematic review assessing the relationship between FSSs and clinical outcomes after primary total hip arthroplasty (THA), total knee arthroplasty (TKA), and unicompartmental knee arthroplasty (UKA).

**Methods:**

The PubMed and Web of Science databases were queried from January 1955 through December 2021 for studies investigating the impact of at least one FSS (fibromyalgia, irritable bowel syndrome (IBS), chronic headaches, and chronic low back pain) on outcomes after primary THA/TKA/UKA. Outcomes of interest included patient-reported outcome measures (PROMs), postoperative opioid use, complications, revisions, and costs of care.

**Results:**

There were twenty-eight studies, including 768,909 patients, of which 378,384 had an FSS. Five studies reported preoperative PROMs prior to THA/TKA, all of which showed worse PROMs among patients with at least 1 FSS diagnosis. Thirteen studies reported postoperative PROMs after THA/TKA, all of which demonstrated worse PROMs among patients with at least 1 FSS diagnosis. Patients with FSS diagnoses were more likely to continue using opioids at 3, 6, and 12 months following TKA, THA, and UKA. Medical and surgical complications, as well as revision rates, were higher among patients with FSSs.

**Conclusion:**

Patients with FSSs have inferior PROMs and are at increased risk for prolonged postoperative opioid use, medical and surgical complications, and revision after hip and knee arthroplasty. Improved understanding of the factors influencing the success of hip and knee arthroplasty is critical. Future studies should address the biopsychosocial determinants of health that can impact outcomes after total joint arthroplasty.

## Introduction

The volume of total joint arthroplasty (TJA) procedures performed in the USA continues to rise on an annual basis [1, 2]. Projections indicate that total hip arthroplasty (THA) and total knee arthroplasty (TKA) volume will increase up to 145% and 147%, respectively, over the next decade [2]. Between 1.9 and 2.6 million TJA procedures are expected to be performed in the year 2030 [2]. Total joint arthroplasty consistently improves patient quality of life with excellent overall results and low complication rates [3–8]. Despite their generally high rates of success, up to 7% and 20% of patients remain dissatisfied following THA and TKA, respectively [9, 10]. Various patient-specific factors, including social and demographic characteristics, medical comorbidities, and mental health disorders, have been shown to influence outcomes following TJA [11–15].

Functional somatic syndromes (FSS) have been defined as “several related syndromes that are characterized more by symptoms, suffering, and disability than by disease-specific, demonstrable abnormalities of structure or function” [16]. Examples of FSSs include fibromyalgia, irritable bowel syndrome, chronic headaches, chronic fatigue syndrome, and chronic low-back pain [16–21]. Given the interrelatedness of these conditions with high rates of co-occurrence and overlap in definitions, prior authors have argued that FSSs should be considered as a single condition rather than multiple disorders [21]. FSSs have received increasing attention within the medical communities as they can result in significant disability, psychological distress in patients, and disproportionate use of health care resources [21–24]. FSSs have been associated with poor outcomes and higher hospitalization costs following shoulder arthroplasty [25]. However, the relationship between FSSs and outcomes following total joint arthroplasty is not clear and has not been systematically reviewed.

With an increased emphasis on patient-reported outcomes, an enhanced understanding of the factors influencing success following TJA is imperative. The primary purpose of this systematic review was to assess the relationship between functional somatic syndromes and patient-reported outcome measures (PROMs) after primary hip and knee arthroplasty. Secondary outcomes included postoperative opioid consumption, postoperative complications, revision or re-operation, and costs of care.

## Materials and methods

This systematic review was conducted and reported in adherence to the 2020 Preferred Reporting Items for Systematic Reviews and Meta-Analyses (PRISMA) Statement.

### Search strategy

Relevant randomized control trials (RCTs) and retrospective or prospective cohort studies that examined the relationship between at least one FSS and orthopedic clinical outcomes following hip and/or knee arthroplasty were acquired through a comprehensive electronic literature search in two databases (PubMed and Web of Science) from January 1955 to December 2021.

The search strategy was: (“fibromyalgia” OR “functional somatic syndrome” OR “irritable bowel syndrome” OR “chronic headaches” OR “chronic migraines” OR “chronic low back pain”) AND (“hip arthro*” OR “knee arthro*” OR “hip replacement” OR “knee replacement”).

### Selection of studies

Two authors (K.M., R.M.) independently screened and assessed the titles, abstracts, and full text of retrieved literature for their eligibility of inclusion and excluded any irrelevant studies and/or duplicates. The two authors also searched the reference lists of identified studies for potential inclusion. Any discrepancies regarding the inclusion and/or exclusion of a given study were to be resolved by discussion among the two authors (K.M., R.M.) and the corresponding author (M.J.S.); however, no discrepancies occurred during the selection process.

### Inclusion and exclusion criteria

For this systematic review, articles were included if they (1) were written in English language; (2) involved subjects who underwent primary hip or knee arthroplasty (TKA, UKA, or THA); (3) compared outcomes of patients with pre-treatment diagnosis of at least one of four well-recognized FSSs (fibromyalgia, irritable bowel syndrome, chronic headaches, low back pain) to patients without a pre-treatment diagnosis of any of the aforementioned FSSs. All other studies were excluded if they did not include patients who underwent primary hip or knee arthroplasty, did not include patients with the aforementioned FSSs, and did not compare outcomes of patients with at least one of the included FSSs to patients without a pre-treatment diagnosis were excluded.

### Types of outcome measures

The primary outcome measures of this systematic review were baseline and postoperative patient-reported outcome measures of pain and function. Secondary outcome measures included postoperative opioid consumption, postoperative complication rates, revision or reoperation, and costs of care.

### Data extraction

Data pertaining to patient demographics (age, sample size, gender), type of procedure, follow-up, loss to follow-up, PROMs, postoperative opioid use, complication rates, revision rates, and hospitalization costs were recorded.

### Quality assessment

Evaluation of risk of bias was performed using the Methodological Index for Non-Randomized Studies (MINORS) criteria. The MINORS criteria is a validated tool that contains 12 items, each scoring from 0–2. The maximum score for non-comparative studies is 16 and the maximum score for comparative studies is 24.

## Results

### Study identification

**Fig. 1:**
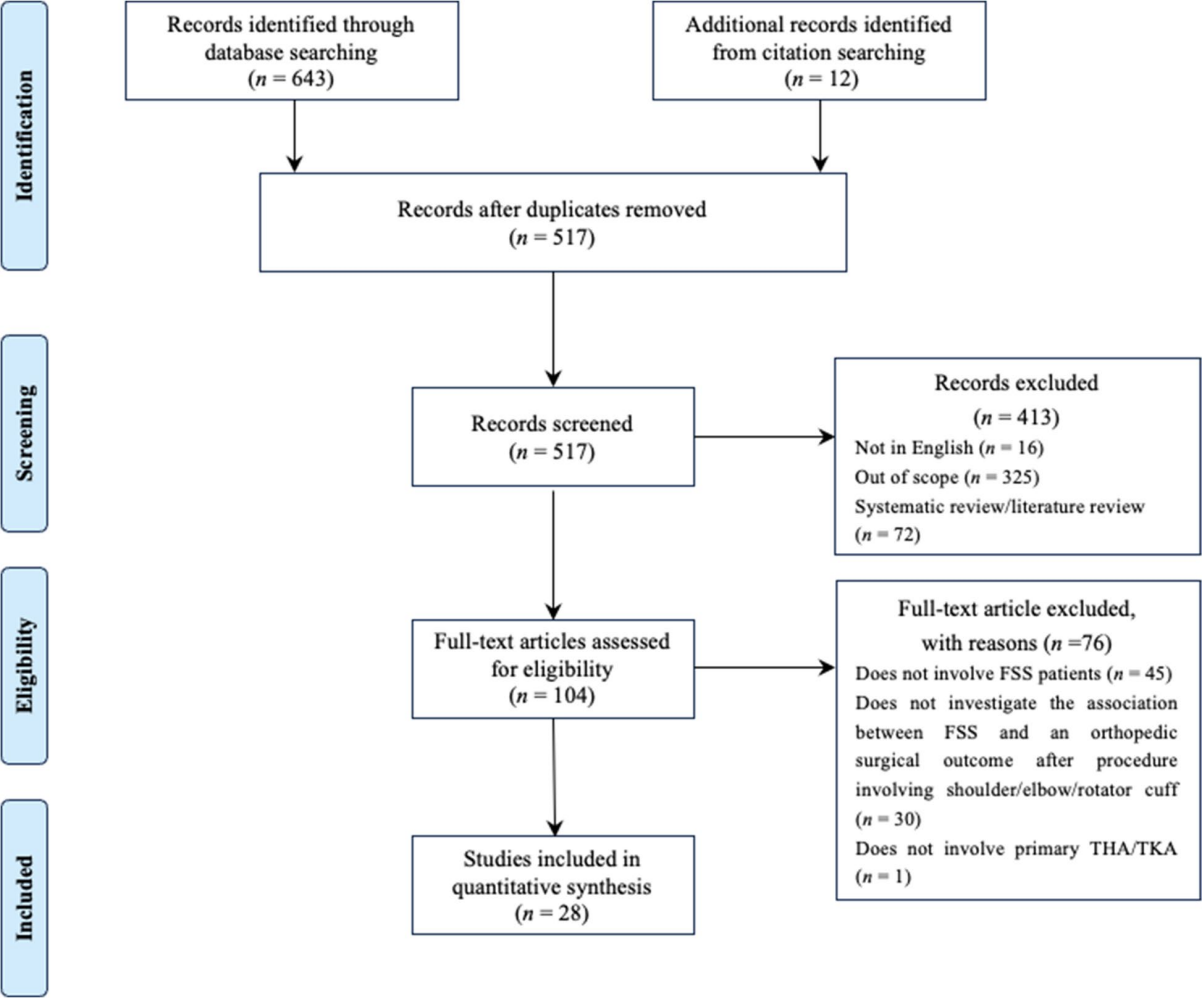
PRISMA flow diagram depicting study selection for systematic review

The search identified a total of 517 studies, of which 28 met the inclusion criteria. 12 of these studies were identified through citation searching. A PRISMA flow diagram is shown in Fig. 1.

### Study characteristics

There were 17 retrospective cohort studies [26–42], 10 prospective cohort studies [43–52], and 1 case–control study [53]. The included studies had a mean MINORS score of 16.9 out of 24 for comparative studies and 10 out of 16 for the 1 non-comparative study. Sixteen studies were conducted in the USA, with the remainder conducted in UK, Scotland, Spain, Australia, Canada, and Denmark. Sample sizes ranged from 75 to 305,510 subjects. Eighteen studies were level III evidence and 10 studies were level II evidence. A summary of study characteristics is presented in Table 1.

**Table 1:** Summary of all studies included in the systematic review

Author, year	Country	Study design	No. of subjects	Surgery	Determination of FSS	Baseline FSS characteristics (*n*)	Outcome measures	Follow-up (mean)
Ayers, 2013 [43]	USA	prospective cohort	180	TKA	Oswestry questionnaire	Low back pain (89)	1) SF-36 physical functioning	6 months
Bedard, 2017 [26]	USA	retrospective cohort	37,393	THA	medical record	Myalgia (5,332); back pain (17,787)	1) postoperative opioid refills	1 year
Bedard, 2017 [27]	USA	retrospective cohort	73,959	TKA	medical record	Myalgia (10,137); back pain (27,705)	1) postoperative opioid refills	1 year
Bedard, 2018 [35]	USA	retrospective cohort	4,205	UKA	medical record	Myalgia (534); back pain (1,419)	1) postoperative opioid refills	1 year
Bican, 2011 [53]	USA	case control (matched)	118	TKA	medical record	Fibromyalgia (59)	1) postoperative satisfaction; 2) SF-36	3.4 years
Boyle, 2014 [36]	UK	retrospective cohort (matched)	345	TKA	medical record	Low back pain (40)	1) OKS; 2) American KSS; 3) SF-12	2 years
Brummett, 2013 [44]	USA	prospective cohort	519 (233 TKA, 286 THA)	TKA, THA	2011 ACR survey criteria	High FMSS (147); Moderate FMSS (199); Low FMSS (170)	1) postoperative opioid use	NA
Cancienne, 2018 [37]	USA	retrospective cohort	113,337	TKA	medical record	Fibromyalgia (24,920); back pain (69,908); migraines (7,753)	1) prolonged postoperative narcotic use	1 year
Clement, 2013 [45]	UK	prospective cohort	2,392	TKA	self-reported	Low back pain (829)	1) OKS; 2) SF-12; 3) patient satisfaction	1 year
Clement, 2018 [38]	UK	retrospective cohort	2,589	TKA	self-reported	Low back pain (1,309)	1) knee stiffness	1 year
Collados-Maestre, 2016 [46]	Spain	prospective cohort (matched)	144	TKA	medical record, self-reported	Low back pain (48)	1) SF-12; 2) WOMAC; 3) VAS for satisfaction; 4) KSS	min. of 2 years
Cregar, 2021 [39]	USA	retrospective cohort	106,874	TKA	medical record	Fibromyalgia (15,419)	1) LOA (1 year); 2) Revision (2 years)	min. of 2 years
D’Apuzzo, 2012 [40]	USA	retrospective cohort	110	TKA	medical record	Fibromyalgia (110)	1) American KSS; 2) Surgical complications; 3) Revision rate	7 years
Escobar, 2007 [47]	Spain	prospective cohort	640	TKA	self-reported from a questionnaire	Low back pain (411)	1) WOMAC; 2) SF-36	6 months
Hansen, 2017 [41]	Australia	retrospective cohort	15,020	TKA	medical record	Low back pain (349)	1) chronic opioid use	1 year
Inacio, 2016 [42]	Australia	retrospective cohort	9,525	THA	medical record	Migraines (28); back pain (1,367)	1) chronic opioid use	1 year
Kim, 2017 [28]	USA	retrospective cohort	57,545 (39,418 TKA, 18,127 THA)	TKA, THA	medical record	Fibromyaglia (4,719); back pain (23,478); migraines (4,546)	1) persistent opioid use	1 year
Loth, 2017 [29]	UK	retrospective cohort	251	THA	self-reported	Low back pain (135)	1) OHS; 2) FJS-12; 3) SF-12	1 year
Mehta, 2015 [48]	Canada	prospective cohort	494	TKA	self-reported (survey)	Low back pain (399)	1) Pain and Function/Daily Activity KOOS subscales	1 year
Moore, 2019 (a) [31]	USA	retrospective cohort (matched)	305,510	TKA	medical record	Fibromyalgia (152,755)	1) readmission rates; 2) total costs; 3) total reimbursements; 4) net losses for surgical and medical complications	90 days
Moore, 2019 (b) [30]	USA	retrospective cohort (matched)	305,510	TKA	medical record	Fibromyalgia (152,755)	1) Medical complications	90 days
Namba, 2018 [32]	USA	retrospective cohort	23,726	TKA	medical record	Fibromyalgia (381); back pain (2,754); migraines (332); chronic tension headaches (124)	1) number of postoperative opioid prescriptions	1 year
Prentice, 2019 [49]	USA	retrospective cohort	12,560	THA	medical record	Fibromyalgia (122); back pain (2,248); migraines (123); chronic tension headaches (54)	1) number of postoperative opioid prescriptions	1 year
Quintana, 2009 [50]	Spain	prospective cohort	788	THA	medical record	Back pain (402)	1) SF-36; 2) WOMAC	2 years
Sheth, 2020 [51]	USA	prospective cohort	258	TKA, THA	2011 ACR survey criteria, medical record	Fibromyalgia (8); back pain (61)	1) prolonged opioid use	90 days
Skrejborg, 2019 [33]	Denmark	consecutive cohort	352	TKA	medical record	Fibromyalgia (6)	1) NRS for pain	5 years
Sodhi, 2019 [34]	USA	retrospective cohort (matched)	305,510	TKA	medical record	Fibromyalgia (152,755)	1) Surgical complications	NA
Sullivan, 2009 [52]	Canada	prospective cohort	75	TKA	questionnaire, self-reported	Back pain (37)	1) WOMAC	6 weeks

*Abbreviations*: *THA* Total hip arthroplasty, *TKA* Total knee arthroplasty, *SF-36* 36-Item Short Form Survey, *iHOT-33* International Hip Outcomes Tool, *HOS-ADL* Hip Outcome Score Activities of Daily Living Subscale, *OKS* Oxford Knee Scores, *KSS* Knee Society Scores, *WOMAC* Western Ontario and McMaster Universities Osteoarthritis Index, *BPI* Brief Pain Inventory, *LOA* Lysis of adhesion, *SANE* Single Assessment Numeric Evaluation, *VAS* Visual Analogue Scale, *FJS-12* Forgotten Joint Score-12, *KOOS* Knee Injury and Osteoarthritis Outcome Score, *LEAS* Lower extremity activity scale, *NRS* Numeric rating scale, *TKA* Total knee arthroplasty, *THA* Total hip arthroplasty *ACR* American College of Rheumatology, *FMSS* Fibromyalgia survey score

### Study findings

The total number of subjects was 768,909 (Table 2). Twenty studies assessed chronic low back pain, 16 studies assessed fibromyalgia, and 5 assessed chronic migraines/tension headaches. Medical records (14) and American College of Rheumatology (ACR) criteria (2) were used to assess for fibromyalgia. Medical records (12), self-report/questionnaires (7), and a combination of self-report/questionnaires and medical records (1) were used to determine the presence of low back pain. Medical records (5) were used to determine the presence of chronic migraines/tension headaches. Nineteen studies involved outcomes after primary TKA, 5 studies involved outcomes after primary THA, and 3 involved both TKA and THA. One study reported outcomes following unicompartmental knee arthroplasty (UKA). Minimum follow-up lasted for 6 weeks and maximum follow-up for 7 years. Using the 15 studies that reported mean age, the weighted mean age of patients was 62.5 years. 67.7% (*n* = 520,421) of the subjects were female.

**Table 2:** Patient demographic characteristics and level of evidence of all included studies

Variable	Value
Total Patients, No	768,909
Total Patients with FSS, No	378,384
Fibromyalgia, No	198,646
Studies with fibromyalgia patients, No	13
Chronic Back Pain, No	150,775
Studies with chronic back pain patients, No	20
Myalgia, No	16,003
Studies with myalgia patients, No	3
Chronic Migraines or Headaches, No	12,960
Studies with migraine/headache patients, No	5
Age, mean, year	62.47
Time to Follow-up, mean, mo	17.67
Level of Evidence, No	
I	0
II	10
III	18
IV	0

Mean age was calculated from studies that reported mean age as a continuous variable

“Total Patients, No.” reflects both total experimental and control group patients across all studies

### Patient-reported outcome measures

Four studies (*n* = 2,999) reported baseline PROMs before TKA. All four studies reported no significant difference in preoperative scores when comparing patients with at least one FSS to controls. One study (*n* = 251) reported baseline PROMs before THA and found that patients with at least one FSS had lower preoperative PROMs as compared to controls.

Thirteen studies (*n* = 8,478) reported postoperative PROMs (Table 3)*.* PROMs reported included 36-item Short Form Health Survey (SF-36), 12-item Short Form Survey (SF-12), Oxford Knee Scores (OKS), American Knee Society Scores (AKSS), Oxford Hip Score (OHS), Western Ontario and McMaster Universities Score (WOMAC), Visual Analogue Scale (VAS), Forgotten Joint Score-12 (FJS-12), Knee Injury and Osteoarthritis Outcome Score (KOOS), Numeric Rating Scale (NRS), pain, and satisfaction.

**Table 3:** Patient reported outcome measures (PROMs) following total joint arthroplasty among patients with functional somatic syndromes

Author, Year	Surgery	PROM(s)	Results
Ayers, 2013 [43]	TKA	SF-36 (PF)	OR = -3.68^a^ (Mild), -6.52^a^ (Moderate), -7.31^a^ (Severe)
Bican, 2011 [53]	TKA	Likert Satisfaction Scale	-12.44^a^
		SF-36 (PF)	-16.79^a^
		SF-36 (MH)	-15.8^a^
Boyle, 2014 [36]	TKA	OKS	31^a^
		AKSS	152^a^
		SF-12 (PC)	34^a^
		SF-12 (MC)	48^a^
Clement, 2013 [45]	TKA	Patient satisfaction	OR = 0.66^a^ (back pain vs. Control)
		OKS	31.3^a^
		SF-12 (PC)	35.5^a^
		SF-12 (MC)	48.5^a^
Clement, 2018 [38]	TKA	Postoperative Stiffness	OR = 1.81^a^ (Back pain vs. Control)
Collados-Maestre, 2016 [46]	TKA	WOMAC (Pain)	73.2^a^
		WOMAC (Function)	71.4^a^
		ODI	4^a^ (Low), 26^a^ (Moderate), 18^a^ (Severe)
		AKSS (Knee)	82.9^a^
		AKSS (Function)	81.8^a^
		SF-12 (PC)	43.7^a^
		SF-12 (MC)	31.2^a^
		VAS	64.5^a^
D'Apuzzo, 2012 [40]	TKA	AKSS	84^a^ (Pre vs. Post-Op)
Escobar, 2007 [47]	TKA	SF-36 (PF)	1.19
		SF-36 (MH)	3.64^a^
		WOMAC (Pain)	-5.26^a^
		WOMAC (Function)	-4.26^a^
		WOMAC (Stiffness)	-6.20^a^
Loth, 2017 [29]	THA	OHS	16.5^a^
		FJS-12	35.6^a^
		SF-12 (PC)	39.2^a^
		SF-12 (MC)	46.9^a^
Mehta, 2015 [48]	TKA	KOOS (Pain)	ß = 7.46^a^ (6 months), 6.86^a^ (1 year)
		KOOS (Function)	ß = 7.63^a^ (6 months), 5.96^a^ (1 year)
Quintana, 2009 [50]	THA	WOMAC (Pain)	ß = 2.28 (6 months), -5.32^a^ (2 year)
		WOMAC (Function)	ß = 2.36 (6 months), -7.23^a^ (2 year)
		WOMAC (Stiffness)	ß = 0.68 (6 months), -4.94^a^ (2 year)
		SF-36 (PF)	ß = -3.12 (6 months), -7.38^a^ (2 year)
		SF-36 (MC)	ß = -2.87^a^ (6 months), -0.81^a^ (2 year)
Skrejborg, 2019 [33]	TKA	Numeric Rating Scale	OR = 20.66^a^ (FM vs. Control)
Sullivan, 2009 [52]	TKA	WOMAC (Pain)	0.11
		WOMAC (Function)	0.23^a^

• *SF-36 (PF)* Short Form 36 Physical Functioning, *SF-36 (MH)* Short Form 36 Mental Health, *SF-12 PC* Short Form 12 Physical Component, *SF-12 MC*, Short Form 12 Mental Component, *iHOT-33* International Hip Outcome Tool 33, *HOS-ADL* Hip Outcome Score (HOS) Activities of Daily Living (ADL), *OKS* Oxford Knee Score, *AKSS* American Knee Society Score, *WOMAC* Western Ontario and McMaster Universities Osteoarthritis Index (3 components – Pain, Function, Stiffness), ODI Oswestry Disability Index, *VAS* Visual Analogue Scale, *OHS* Oxford Hip Score, *FJS-12* Forgotten Join Score 12, *LEAS* Lower Extremity Activity Scale, *KOOS* Knee Injury & Osteoarthritis Score

• All results reflect postoperative outcomes

• Data is given as the mean result of the FSS group and represents the statistical difference between the FSS group and control group, unless otherwise stated

• Ayers (2013) six-month postoperative SF-36 PF scores are reported as odds ratios comparing each level of preoperative low back pain (mild, moderate, severe), determined by preoperative ODI scores, to a reference group (no low back pain)

• Bican (2011) and Escobar (2007) reported results as mean differences

• Boyle (2014) reported results as median

• Collados-Maestre (2016) ODI results are stratified by low back pain survey score: low, moderate, and severe

• D'Apuzzo (2012) reported postoperative outcomes within the respective FSS group and are tested for statistical difference with pre-operative FSS group values

• Mehta (2015), Quintana (2009) and Sullivan (2009) reported results as beta coefficients (ß) from linear regression analysis

• Sullivan (2009) reported outcomes as a beta coefficient as per linear regression analysis

^a^Statistically significant (*P* < 0.05)

Overall, SF-36 Physical Functioning Scores (PFS), SF-12 scores, OKS, AKSS, WOMAC, satisfaction, and NRS for pain were all worse among patients undergoing TKA with at least one FSS in comparison to controls. Two studies reported SF-36 scores following TKA and found that patients with at least one FSS had worse Physical Functioning Scores (PFS) than patients without at least one FSS. Three studies examined SF-12 scores after TKA and reported that patients with at least one FSS had worse scores than controls. Oxford Knee Scores and American Knee Society Scores following TKA were each reported by two studies. In comparison to controls, patients with at least one FSS demonstrated worse outcome measures with both of these scoring systems. Following TKA, patients with at least one FSS showed worse WOMAC scores compared to controls in 4 studies. One study reported KOOS following TKA and found that patients with an FSS diagnosis had worse outcomes. In terms of postoperative satisfaction, 3 studies reported that patients with an FSS diagnosis were significantly less satisfied than controls. In terms of postoperative pain metrics, one study demonstrated that patients with at least one FSS had worse NRS for pain than patients without FSS following TKA.

SF-36 physical functioning scores, SF-12 scores, WOMAC, OHS, and FJS-12 scores were reported following THA. Loth et al. (2017) found that patients with at least one FSS had worse SF-12, OHS, and FJS-12 scores following THA compared to controls [29]. Quintana et al. (2009) reported that patients with an FSS diagnosis had worse SF-36 PFS and WOMAC scores following THA than controls [50].

### Opioid use

**Fig. 2:**
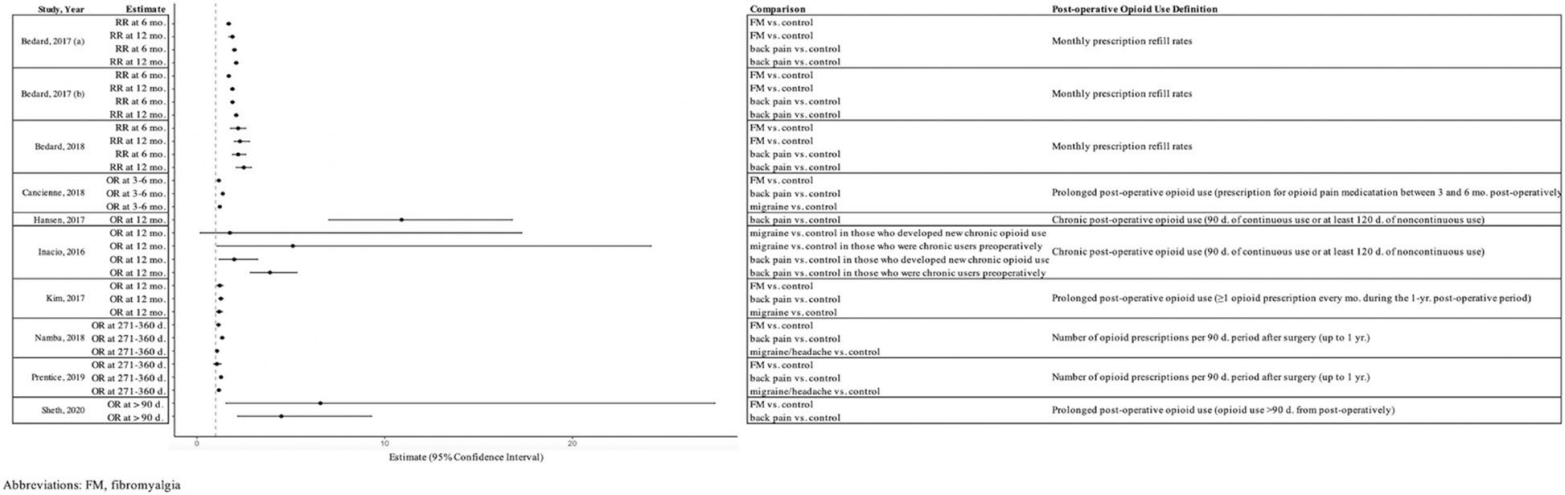
Visual representation of post-operative opioid use following total joint arthroplasty among patients with functional somatic syndromes

Eleven studies (*n* = 348,047) reported postoperative opioid use following TJA. (Fig. 2) Several studies reported postoperative opioid use at many time points. However, we chose representative time points to summarize in Fig. 2. All 11 studies concluded that patients with at least one FSS are at risk for higher opioid use postoperatively. Higher patient ACR fibromyalgia score was associated with greater inpatient opioid consumption following THA and TKA in one study. Patients with FSS diagnoses were more likely to continue using opioids at 3, 6, and 12 months after TKA, THA, and UKA. Chronic back pain and migraine headaches were also shown to be significant risk factors for new chronic opioid use following THA.

### Complications

Four studies (*n* = 412,494) reported postoperative complications (Table 4). Moore et al. (2019) showed that patients with fibromyalgia were 1.95 times more likely to develop any medical complication after TKA than controls (OR = 1.95, 95% CI: 1.86–2.04) [31]. In terms of reoperation, patients with fibromyalgia were significantly more likely to undergo lysis of adhesions for arthrofibrosis within one year after TKA in one study. In the same study, patients with fibromyalgia were more likely to undergo revision TKA within 2 years of index TKA than controls. D’Appuzo et al. (2012) reported an overall revision rate of 6% among patients with fibromyalgia who underwent primary TKA with a mean follow-up of 7 years [40]. Sodhi et al. (2019) showed that patients with fibromyalgia had significantly increased odds of surgical complications following TKA, including bearing wear, periprosthetic osteolysis, mechanical loosening, infection, dislocation, and revision of tibial insert [34].

**Table 4:** Medical and surgical complications including need for revision following total joint arthroplasty among patients with functional somatic syndromes

Author, year	Outcome	Post-intervention
Cregar, 2021 [39]	Lysis of adhesion (LOA) within 1 year post-operatively	OR: 1.10 (0.87–1.39) for FM vs. controls (Medicare database) OR: 1.30 (1.01–1.70)^a^ for FM vs. controls (Humana database)
	Incidence of revision TKA w/in 2 years of index TKA	OR: 1.19 (1.06–1.34)^a^ for FM vs controls (Medicare database) OR: 1.19 (0.93–1.52)^a^ for FM vs controls (Humana database)
D'Apuzzo, 2012 [40]	Incidence of postoperative surgical complications	FM: 13 (9%) arthrofibrosis; 12 (9%) instability; 5 (4%) wound problems; 1 (< 1%) periprosthetic fracture; 1 (< 1%) quad tendon rupture
	Revision rate	FM: 6%
Moore, 2019 [30]	Likelihood of developing any medical complication	OR = 1.95 (1.86–2.04)^a^ for FM vs controls
Sodhi, 2019 [34]	Any surgical complication	FM: 14,416^a^ Control: 9,595^a^ OR = 1.55 (1.51–1.60)^a^
	Articular bearing surface wear of prosthetic joint	FM: 95^a^ Control: 45^a^ OR = 2.11 (1.51–1.60)^a^
	Periprosthetic osteolysis	FM: 53^a^ Control: 31^a^ OR = 1.71 (1.10–2.66)^a^
	Revision of total knee replacement tibial insert (liner)	FM: 113^a^ Control: 74^a^ OR = 1.5 (1.14–2.05)^a^
	Revision of knee replacement total (all components)	FM: 45 Control: 33 OR = 1.36 (0.87–2.14)
	Mechanical loosening of prosthetic joint	FM: 957^a^ Control: 692^a^ OR = 1.34 (1.26–1.53)^a^
	Infection/inflammation	FM: 957^a^ Control: 692^a^ OR = 1.34 (1.26–1.53)^a^
	Dislocation of prosthetic joint	FM: 919^a^ Control: 691^a^ OR = 1.33 (1.21–1.47)^a^
	Periprosthetic fracture around prosthetic joint	FM: 344 Control: 295 OR = 1.17 (0.99–1.36)
	Revision of knee replacement tibial component	FM: 35 Control: 30 OR = 1.17 (0.72–1.90)
	Removal of (cement) spacer	FM: 28 Control: 26 OR = 1.08 (0.63–1.84)
	Broken prosthetic joint implant	FM: 259 Control: 262 OR = 1 (0.83–1.17)
	Revision of knee replacement patellar component	FM: 26 Control: 37 OR = 0.7 (0.43–1.16)
	Revision of knee replacement not otherwise specified	FM: 35 Control: 50 OR = 0.7 (0.45–1.08)
	Revision of knee replacement femoral component	too few to identify
	Other	FM: 8329^a^ Control: 4907^a^ OR = 1.74 (1.68–1.80)^a^

*Abbreviations*: *LOA* Lysis of adhesion, *TKA* Total knee arthroplasty, *FM* Fibromyalgia

^a^Statistically significant (*P* < 0.05)

### Costs

One study (*n* = 305,510) reported surgical and medical costs among patients who underwent TKA. While patients with fibromyalgia had lower readmission costs than patients without, they incurred greater management costs for medical and surgical complications following total knee arthroplasty in comparison to patients without fibromyalgia, thus resulting in net losses.

## Discussion

Functional somatic syndromes (FSS) are a group of interrelated conditions characterized by the presence of chronic symptoms that cannot be attributed to a known somatic disease or disorder [16, 17]. FSSs are common and have been reported to account for as high as 35% of primary care visits [54, 55]. The etiology of FSSs is not clear; however, they are considered to arise via a complex interaction among biological and psychosocial factors [16, 56]. Common examples of FSSs are fibromyalgia, irritable bowel syndrome, chronic headaches, chronic fatigue syndrome, and chronic low back pain [16–21]. However, prior authors have proposed that FSSs be considered collectively as “variants of a common biopsychosocial process”, given their high rates of co-occurrence and overlap of symptoms [16–21]. Moreover, there is evidence to support that FSSs should be considered on a continuum, as patients with more functional symptoms demonstrate increasing disease severity states [29, 57, 58]. Prior studies have reported the role of individual conditions and isolated functional symptoms in outcomes following total joint arthroplasty [36, 53]. However, the relationship between FSS as a single entity and results following TJA has not been studied, highlighting the importance of our systematic review.

The primary focus of this systematic review was to determine the relationship between FSSs and patient-reported outcome measures following primary hip and knee arthroplasty. Overall, our results showed that the presence of at least one FSS is associated with worse PROMs among patients undergoing hip or knee arthroplasty. Scores for SF-36 or its abbreviated version (SF-12) were reported by 6 studies for patients after TKA and by 2 studies after THA [29, 36, 43, 45, 46, 50, 53]. All of these studies reported worse scores among patients with an FSS diagnosis in comparison to controls. These metrics are typically utilized to assess a patient’s quality of life and comprised of eight health domains, including limitations in physical and social activities, bodily pain, mental health, fatigue, and general health perceptions [59]. The prevalence of psychological disorders such as depression and anxiety is much higher in patients with FSS than in the general population, which certainly could lead to lower scores on the overall SF-36 and SF-12 metrics [17]. However, Ayers et al. (2013) and Bican et al. (2011) specifically reported SF-36 Physical Functioning Scores (PFS) following TKA and demonstrated worse results among patients with at least one FSS [43, 53]. Quintana et al. (2009) showed similar results for THA [50]. In terms of PROMs specific to the hip and knee, OHS, OKS, AKSS, KOOS, and WOMAC were all included in various studies [29, 36, 45–48, 50, 52]. Patients with FSS performed worse on each of these metrics in comparison to controls, even when preoperative scores were not significantly different. The relationship between FSS and PROMs has been previously studied in the orthopedic literature with reference to shoulder arthroplasty. Moverman et al. (2021) found that at least one FSS was associated with both worse 2-year postoperative American Shoulder and Elbow Surgeons Shoulder (ASES) and Single Assessment Numerical Evaluation (SANE) scores [25]. Furthermore, this study demonstrated that each additional functional disorder was associated with a stepwise decline in these PROMs. In light of FSS as a continuum, future prospective studies are needed to investigate the impact of an increasing number of functional disorders on outcomes following total hip and knee arthroplasty.

In addition to PROMs, we systematically reviewed the impact of FSSs on postoperative opioid consumption following hip and knee arthroplasty. Increased inpatient opioid consumption has been shown to be strongly associated with an increased risk of chronic postoperative opioid use following TJA [60]. Brummett et al. (2013) reported that higher patient scores on the American College of Rheumatology (ACR) criteria for fibromyalgia were independently associated with increased inpatient postoperative opioid consumption following THA and TKA [44]. FSS diagnoses appear to increase the risk of prolonged postoperative opioid use up to 1 year after TJA. Bedard et al. (2017) found that both fibromyalgia and chronic back pain significantly increased the risk of continued opioid use at 6 and 12 months following total hip arthroplasty [26]. Similar results were reported following total knee and unicompartmental knee arthroplasty [27, 35]. Multiple additional studies supported these findings, with fibromyalgia, chronic back pain, and chronic headaches increasing the risk of opioid prescription refills and protracted opioid use following TJA [28, 32, 37, 41, 49, 51]. Preoperative opioid use has been shown to increase the risk of chronic postoperative opioid use following hip and knee arthroplasty [49, 60–65]. A study by Agger et al. (2018) reported that prescription opioids are used by 26% of patients with multiple functional somatic syndromes at baseline [66]. However, FSSs might also increase the risk of new persistent opioid use after TJA among patients who were not previously opioid users [42]. Given the ongoing opioid epidemic within the USA, an improved recognition of the risk factors for prolonged opioid use following TJA is crucial. Preoperative patient education and counseling regarding postoperative pain control expectations among patients with FSSs undergoing TJA is critical. Furthermore, detailed multimodal pain control plans (potentially including regional anesthesia and non-opioid analgesics) should be formulated for these patients.

Medical complications following total joint arthroplasty increase patient morbidity and can lead to higher episode-of-care costs [67]. Moore et al. (2019) performed a retrospective review of the PearlDiver database and found that, compared to propensity score matched controls, patients with fibromyalgia had increased odds of developing any medical complication after TKA [30]. Our systematic review also demonstrates that FSSs are a risk factor for surgical complications and revision following primary hip and knee arthroplasty. In an analysis of both the Humana and Medicare databases, Cregar et al. (2021) showed that fibromyalgia was associated with an increased risk of undergoing lysis of adhesions for arthrofibrosis within 1 year of primary TKA as well as revision TKA within 2 years [39]. D’Appuzo et al. (2012) reported an overall revision rate of 6% among patients with fibromyalgia who underwent primary TKA with a mean follow-up of 7 years [40]. This is consistent with previously published registry data reporting overall 10-year TKA revision rates between 4.9 and 7.8% [68]. However, Sodhi et al. (2019) found that patients with fibromyalgia had a significantly increased risk of surgical complications following TKA such as bearing wear, periprosthetic osteolysis, mechanical loosening, infection, dislocation, and tibial insert revision [34].

Functional somatic syndromes are associated with high healthcare utilization rates and total annual healthcare costs [69–73]. Patients with FSSs have been shown to incur higher hospitalization costs following shoulder arthroplasty [25]. In a retrospective database study by Moore et al. (2019), patients with fibromyalgia incurred greater management costs for medical and surgical complications following total knee arthroplasty in comparison to patients without fibromyalgia [31]. Within bundled payment reimbursement models, healthcare providers are responsible for all costs incurred during an episode of care. Given the evidence that FSSs can increase episode-of-care costs, perhaps adjusted target prices are needed for these patients when undergoing total hip and knee arthroplasty.

Inferior outcomes following TJA among patients with FSSs may be circumvented by managing these conditions preoperatively. Literature has shown that using a biopsychosocial, patient-involving approach is an effective management strategy [74]. In addition, FSSs can be managed using a multi-modal approach, including diagnosis explanation, guided self-help, cognitive behavioral therapy, and specialist referral [75].

We recognize limitations to our analysis. Due to the nature of systematic reviews, studies investigating outcomes after hip and knee arthroplasty among patients with FSSs that satisfy our inclusion criteria could have been omitted. However, our search was performed systematically using two separate databases. Furthermore, concern for publication bias exists with any systematic review. In addition, the majority of included studies were cohort studies which are prone to selection bias, confounding factors, and information bias. Further, based on the MINORS criteria, the included studies were not of high quality. We chose to include four of the most well-recognized functional somatic syndromes in our search, but did not search for other more obscure FSSs such as chronic fatigue syndrome or multiple chemical sensitivity. These four FSSs were selected based on the substantial literature regarding these conditions. However, other search terms may have captured additional studies in our review. In addition, follow-up time of the included studies ranged from 6 weeks to 7 years, with most studies having a follow-up time of 1 year or less. Thus, further studies should focus on the outcomes of patients with FSSs following joint arthroplasty after mid- or long-term follow-up.

## Conclusion

In conclusion, the present systematic review demonstrates that patients with FSSs have inferior PROMs and are at increased risk for prolonged postoperative opioid use, medical and surgical complications, and revision after hip and knee arthroplasty. Future studies should address the biopsychosocial determinants of health that can impact outcomes after total joint arthroplasty.

## Data Availability

The data analyzed is available in the publications referenced studies.

## Abbreviations

TJA: Total joint arthroplasty
THA: Total hip arthroplasty
TKA: Total knee arthroplasty
FSSs: Functional somatic syndromes
PROMs: Patient-reported outcome measures
PRISMA: Preferred Reporting Items for Systematic Reviews and Meta-Analyses
RCTs: Randomized controlled trials
MINORS: Methodological Index for Non-Randomized Studies
ACR: American College of Rheumatology
UKA: Unicompartmental knee arthroplasty
SF-36: 36-Item Short Form Health Survey
SF-12: 12-Item Short Form Survey
OKS: Oxford Knee Scores,
AKSS: American Knee Society Scores
OHS: Oxford Hip Score
WOMAC: Western Ontario and McMaster Universities score
VAS: Visual analogue scale
FJS-12: Forgotten Joint Score-12
KOOS: Knee Injury and Osteoarthritis Outcome Score
NRS: Numeric Rating Scale
PFS: Physical Functioning Scores
ASES: American Shoulder and Elbow Surgeons Shoulder
SANE: Single Assessment Numerical Evaluation
